# Supplementary material for: The Anti-Constipation Effect of Bifidobacterium Longum W11 Is Likely Due to a Key Genetic Factor Governing Arabinan Utilization
Source: Microorganisms. 2024 Aug 9;12(8):1626. doi: 10.3390/microorganisms12081626 (PMC11356487; doi:10.3390/microorganisms12081626)
Supplement: Supplementary file 1 [file microorganisms-12-01626-s001.zip › microorganisms-3142546-supplementary.pdf]

Supplementary Table S1.

RNA and DNA quantifications.

**Timepoint**	**Sample**	**Nanodrop**	**Qubit RNA**	**Qubit DNA**
T0	*B. longum* W11 (Ctrl)	383	154	14
T16	*B. longum* W11 (Ctrl)	97	94	11.7
	B. longum W11 (Ara)	42.6	25	11.1
	B. longum W11 (Glu)	39	25.6	11.2
T24	*B. longum* W11 (Ctrl)	96.7	124	17.8
	B. longum W11 (Ara)	132	98.8	22.4
	B. longum W11 (Glu)	118	100	17.5
T48	*B. longum* W11 (Ctrl)	39.7	25.2	1.57
	B. longum W11 (Ara)	171.4	120	10.4
	B. longum W11 (Glu)	244.2	254	16.3

RNA quantification was obtained by using Nanodrop and QUBIT fluorometers. Equal amounts of RNA were then retro transcribed into cDNA for the qPCR analyses and 1 ng of cDNA was used for all qPCR reactions. Ctrl: control; Ara: arabinan; Glu: glucose. Values are expressed as ng/µl.
